# Barred owls and landscape attributes influence territory occupancy of northern spotted owls

**DOI:** 10.1002/jwmg.793

**Published:** 2014-10-09

**Authors:** Stan G Sovern, Eric D Forsman, Gail S Olson, Brian L Biswell, Margaret Taylor, Robert G Anthony

**Affiliations:** 1 Department of Fisheries and Wildlife, Oregon State UniversityCorvallis, OR, 97331, USA; 2 USDA Forest Service, Pacific Northwest Research Station3200 S.W. Jefferson Way, Corvallis, OR, 97331, USA; 3 Oregon Cooperative Fish and Wildlife Research Unit Department of Fisheries and Wildlife Biology, Oregon State UniversityCorvallis, OR, 97331, USA; 4 USDA Forest Service, Pacific Northwest Research Station3625 93rd Avenue S.W., Olympia, WA, 98512, USA

**Keywords:** barred owls, colonization, landscape pattern, local-extinction, northern spotted owls, *Strix occidentalis caurina*, *Strix varia*, territory occupancy modeling, Washington

## Abstract

We used multi-season occupancy analyses to model 2 fates of northern spotted owl territories in relation to habitat amount, habitat fragmentation, and the presence of barred owls in Washington State, USA, 1989–2005. Local colonization is the probability a territory unoccupied by a spotted owl in year *i* would be occupied in year *i* + 1, and local extinction is the probability a territory that was occupied by a spotted owl in year *i* would be unoccupied in year *i* + 1. We found a negative relationship between local extinction probability and amount of late-seral forest edge. We found a negative relationship between colonization probability and the number of late-seral forest patches (higher fragmentation), and a negative relationship between colonization probability and the amount of non-habitat within 600 m of a spotted owl territory center (Akaike weight = 0.59). The presence of barred owls was positively related to extinction probability and negatively related to detection probability of spotted owls. The negative relationship between presence of barred owls and detectability of spotted owls indicated that spotted owls could be modifying their calling behavior in the presence of barred owls. The positive relationship between barred owl detections and local extinction probability suggests that because of competition with barred owls, spotted owls are being displaced. Published 2014. This article is a U.S. Government work and is in the public domain in the USA.

Because of concerns regarding habitat loss, the northern spotted owl (*Strix occidentalis caurina*) has become one of the most intensively studied owls in the world (Noon and Franklin 2002, Anthony et al. 2006, Forsman et al. 2011). Most studies of the habitat relationships of spotted owls indicate that they are associated with late-successional forests (Forsman et al. 1984, 2005; Wiens et al. 2014). In addition, some studies indicate that nest territories of spotted owls tend to be located in areas with comparatively low levels of forest fragmentation (Lehmkuhl and Raphael 1993). Although management in Northwest forests has been altered for decades by efforts to preserve and create spotted owl habitat, spotted owl populations continue to decline (Forsman et al. 2011).

Barred owls (*Strix varia*) were first recorded in Washington State in the early 1960s (Reichard 1974), and have since expanded their range throughout the Pacific Northwest and into California (Taylor and Forsman 1976, Dark et al. 1998, Herter and Hicks 2000, Kelly et al. 2003, Pearson and Livezey 2003, Diller et al. 2014). Barred owls now appear to be competing with northern spotted owls for resources (Hamer et al. 2001, Wiens et al. 2014), displacing spotted owls from historical territories (Hamer 1988, Kelly et al. 2003, Pearson and Livezey 2003, Van Lanen et al. 2011, Wiens et al. 2014), predating spotted owls (Leskiw and Gutiérrez 1998), or hybridizing with them (Kelly and Forsman 2004). The effect of the barred owl invasion is a concern for biologists monitoring spotted owl populations and for management agencies that are trying to manage habitat for spotted owls (Courtney et al. 2004, Buchanan et al. 2007, U.S. Fish and Wildlife Service [USFWS] 2008, Diller et al. 2014).

Several recent studies have explored the use of territory occupancy probabilities of spotted owls as a method for assessing the relative influence of different environmental variables on spotted owls (Dugger et al. 2005, Olson et al. 2005, Kroll et al. 2010, Yackulic et al. 2014). Occupancy modeling is advantageous because animals neither have to be detected during any given survey, nor marked to evaluate environmental relationships to spotted owl presence (MacKenzie et al. 2003). In this study, we used territory occupancy modeling (MacKenzie et al. 2002, 2003; Olson et al. 2005) to assess the influence of habitat and the presence of barred owls on spotted owl territory occupancy the following year.

## STUDY AREA

The Cle Elum Study Area was 1 of 8 long-term demography study areas that was used to monitor population trends of northern spotted owls on federal lands (Lint et al. 1999, Anthony et al. 2006, Forsman et al. 2011). It was located in central Washington State, on the eastern slope of the Cascade Range. Most of the area was mountainous, with deeply incised drainages and ridge tops extending to over 1,500 m. The climate was characterized by warm dry summers and cold winters, and most precipitation occurred as snow in winter. Vegetation was naturally fragmented, with mixed conifer forests of Douglas-fir (*Pseudotsuga menzesii*), grand fir (*Abies grandis*), ponderosa pine (*Pinus ponderosa*), western larch (*Larix occidentalis*), lodgepole pine (*Pinus contorta*), and western white pine (*Pinus monticola*) predominating on north, west, and east aspects, and ponderosa pine forest predominating on southerly aspects. Most forests in the area had also been extensively fragmented by logging and historical wildfires. The ceding of land to railroad developers in the 1800s has resulted in a checkerboard pattern of private and public ownership within the study area (Richardson 1980, Jensen et al. 1995).

## METHODS

### Field Methods

We monitored owls each year in 1989–2005 using a combination of acoustic-lure (callback surveys) and live-lure techniques (Reid et al. 1999). Imperfect detection of spotted owls could be estimated because 1) we conducted repeated surveys with up to 5 visits per territory (Appendix A, available online at www.onlinelibrary.wiley.com) within a survey year and 2) we repeated these surveys for multiple years. We assumed that territory occupancy status was constant throughout the year. This sampling scheme follows the primary and secondary sampling periods described in Pollock (1982). We used only spotted owl calls during surveys, but we recorded all owls that responded, including barred owls.

We banded all spotted owls with unique color bands so they could be identified without recapture (Forsman et al. 1996). The analysis included 88 historical owl territories and 8 polygons that were approximately the same size as a spotted owl territory, but that had no historical or recent detections of spotted owls. We assigned each territory a set of Universal Transverse Mercator (UTM) coordinates to establish the territory center for analyses that included vegetative attributes and distance to barred owl detections. Territory center selection was based on nest tree locations, locations of nestlings, pair locations, female locations, or male locations, in order of precedence, respectively.

In our study, barred owl detections were mostly nocturnal responses to acoustic-lure surveys for spotted owls. We estimated locations of vocalizing barred owls by getting as close to the owl as possible and using visual and auditory clues to estimate the location. We recorded all locations with a hand-held global positioning system (GPS) unit (if the owl was in the immediate vicinity of the surveyor), or by estimating the distance and direction to the owl from a known location and plotting compass azimuths taken on the responding owl plotted on a topographic map (if the owl was not observable). If a territory was not surveyed in a given year, we assigned it a no data code and excluded it from estimates for that year. We conducted this study under the auspices of Oregon State University Institutional Animal Care and Use Committee (IACUC) protocol number 3628 titled “Demography of northern spotted owls in Oregon and Washington.”

### Hypotheses and Model Covariates

The detection probability of spotted owls can vary between good and bad nesting years because nesting owls are more territorial and more sedentary during the nesting season (Anthony et al. 2006). As a result, we included 2 annual reproductive covariates in our analysis of detection probabilities: mean female fecundity (the estimated number of female young produced per female owl assuming a 50:50 sex ratio), and the proportion of females that nested (Appendix B, available online at www.onlinelibrary.wiley.com). We developed a visit-specific covariate that documented whether a barred owl was detected either vocally or visually during any visit to a territory (BAO), and we included a year-specific covariate (BAODIST) for each spotted owl territory that documented whether barred owls were detected within a 0.8-km radius of the territory center (Olson et al. 2005). We used the 0.8-km radius cutoff for BAODIST because Kelly et al. (2003) found that spotted owls were more likely to move to a new location if barred owls were detected within 0.8 km of the historical territory center. We also added 2 time-forward covariates (BAO1 and BAODIST1) to extinction and colonization models. The models with the time-forward covariates use the barred owl detection in year *i* + 1 to affect the extinction or colonization probability estimate for the interval between year *i* and year *i* + 1 (Olson et al. 2005).

#### Cover type map

We developed annual cover type maps of the study area using a geographic information system (ArcGIS; Environmental Systems Research Institute, Inc., Redlands, CA). We combined 23 cover types from a classified Landsat Thematic Mapper image from August 1997 into 3 categories for assessment of vegetation composition, patterns, and fragmentation. Canopy cover categories were 0–39%, 40–69%, and >70%. We divided tree size categories for forested areas into 1–12.9, 13.0–37.9, and ≥38 cm quadratic mean diameter (the diameter of a tree of average basal area) for the non-habitat, mid-seral, and late-seral forest categories, respectively. Given the available cover-type classes on our map, we considered these 3 combinations of cover types to be most relevant to spotted owls based on personal experience. The late-seral conifer category contained closed-canopy cover types generally associated with spotted owl nesting, roosting, and foraging habitat on the study area, whereas the mid-seral conifer and broadleaf category represented forest stands that receive occasional use by owls (Carey et al. 1990). Broadleaf forests represented a very small portion of the habitat on the Cle Elum Study Area and we combined them with the conifer types. The non-forest category contained cover types or canopy cover categories (<40% canopy cover) that we did not consider to be habitat used by spotted owls. We used harvest and disturbance data provided by the Washington State Department of Natural Resources, Forest Practices Division to update the cover map as necessary.

To estimate the accuracy of the cover type map, we compared our map categories to 98 0.05-ha vegetation plots sampled in 1995–1997. Because most of the vegetation plots were taken in closed-canopy forest, we added 102 randomly selected points within the study area to assess the non-habitat type. When the classification of the cover type category was not clear from the plot data alone, we validated the plot data with a 1998 Digital Ortho Quad. Overall classification accuracy of the cover type map versus our test points was 93% using the fuzzy accuracy assessment method of Woodcock and Gopal (2000), where the canopy cover and tree diameter values were within 10% of the cut points to differentiate categories in the map.

#### Landscape metrics

We calculated the proportional coverage of non-habitat and late-seral forest within a radius of 600, 1,500, and 2,400 m around each territory center (Appendix B, available online at www.onlinelibrary.wiley.com). For measures of landscape fragmentation, we extracted 1) the total length of edge between late-seral forest and all other cover types, 2) the total length of edge between non-habitat and all other cover types, 3) the number of patches of late-seral forest, and 4) the mean patch size of late-seral forest in the circle using program FRAGSTATS (McGarigal et al. 2002).

#### Occupancy modeling

For each survey period, we coded the data for a territory as either 1 (spotted owls detected), 0 (spotted owls not detected), or NA (not surveyed). We estimated territory occupancy parameters with a product multinomial likelihood model (MacKenzie et al. 2003, Olson et al 2005:922):

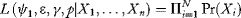
where ψ_1_ is a vector of territory occupancy probabilities for the first primary sampling period, ε and γ are matrices of local extinction and colonization, and *p*_(*i*,*j*)_ is a matrix of detection probabilities of the *i*th occasion in the *j*th season. We used this parameterization to model local extinction and colonization, the processes that determine territory occupancy, as opposed to other parameterizations that can be used to model time-specific territory occupancy directly. We used program MARK (White and Burnham 1999) for all analyses.

#### Hypotheses and model selection

We used a model selection approach to assess the supporting evidence for the following competing hypotheses: 1) the proportion of late-seral forest habitat within a 1,500-m radius of the territory center is positively associated with colonization and negatively associated with extinction; 2) amount of edge and number of late-seral forest patches (as metrics of fragmentation) are negatively associated with colonization and positively associated with extinction; and 3) barred owl presence is negatively associated with detection and colonization, and positively associated with extinction. We also developed a priori models for detection probabilities where detection was constant (.), time-specific (*t*), varied linearly (T), or had a quadratic effect (TT). For within-year time covariates, the order of visits created the time series. We considered models within 2.0 Akaike's Information Criterion (AIC) units of the top model to be competing models (Burnham and Anderson 2002). We used the general model for all other parameters in the model (ψ (.), ε (*t*), γ (*t*)), while testing different hypotheses regarding detection probabilities (Olson et al. 2005).

After we determined the best model(s) for detection probabilities, we then used the structure from that model for detection probabilities and constrained the extinction or colonization parameters by a constant (.), linear (T), or quadratic (TT) time effect. The best model resulting from this suite of constrained extinction or colonization models was the base model for the next step where we replaced the time trend covariates for extinction, colonization, and detection parameters in the base model with one of the barred owl covariates. For detection probabilities, we tested both the BAO and BDIST variables and for extinction and colonization parameters, we tested these variables as well as the time-forward versions (BAO1 and BDIST1). We then added the habitat covariates, 1 per model, to extinction and colonization parameters from the top-ranked model from the barred owl step. After we determined the best habitat covariates for extinction and colonization, we examined some post-hoc models combining the best covariates for habitat composition and pattern. Because the results of our analysis were similar regardless of whether we used pair occupancy (pairs detected) or simple occupancy (≥1 owl detected), we present only the results for the analysis of pair occupancy.

## RESULTS

**Figure 1:**
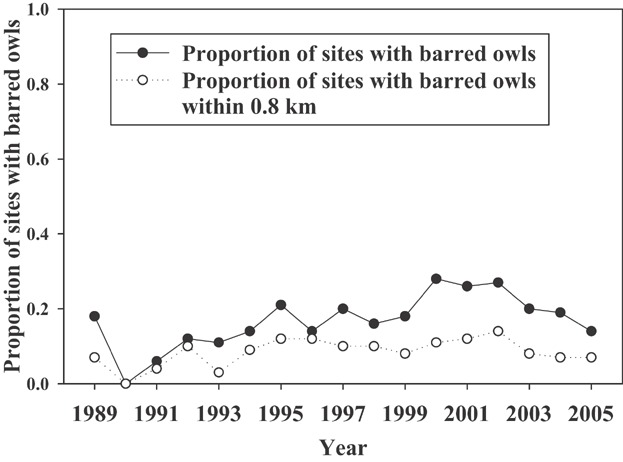
Year-specific proportions of northern spotted owl territories (sites) with barred owl detections within 1,500 m (solid circles) and within 800 m (open circles) of the site center, Cle Elum Study Area, Washington, USA, 1989–2005.

Our average maximum number of visits per territory per year was 5 (Appendix A, available online at www.onlinelibrary.wiley.com). From 1990 to 2005, we detected spotted owls at 92 spotted owl territories. We detected barred owls at 44 of these territories. The annual percentage of spotted owl territories in which we detected barred owls ranged from 0% to 28% (Fig. 1). We found an increase in the probability of detecting a spotted owl at a territory among visits within a survey year, but the trend was different for each year. Among-year spotted owl detection probability did not appear to be related to the mean proportion of owls that nested or fecundity across territories. The probability of detecting a spotted owl varied among years, and the presence of a barred owl at the territory decreased the probability of detecting a spotted owl. We used a post-hoc model that included a combination of among- and within-year detection probabilities from the top 2 AIC-ranked models for detection probability in subsequent modeling (Table1).

**Table 1:** Habitat models used in the analysis of occupancy, including local extinction probability (ε) and local colonization probability (γ), of spotted owl territories on the Cle Elum Study Area, Washington, 1989–2005. Parameter structure for occupancy (ψ) and recapture probability (*p*) was the same in all models {ψ(.), *p* (T_1_ = T_2_ = …T_*i*_, BAO + *t*)}; AIC = Akaike's Information Criterion and *K* = number of parameters

Model^a^	AIC	ΔAIC	AIC weights	*K*
ε(BAO1 + LSFEDGE), γ(TT + NO600 + LSFNP)^b^	2,936.83	0.00	0.59	28
ε(BAO1 + LSFEDGE), γ(TT + LSFNP)^b^	2,938.91	2.08	0.21	27
ε(BAO1), γ(TT + NO600 + LSFNP)^b^	2,939.72	2.89	0.14	27
ε(BAO1), γ(TT + LSFNP)	2,942.68	5.85	0.03	26
ε(BAO1), γ(TT + NO600)	2,944.90	8.06	0.01	26
ε(BAO1), γ(TT + LSF1500)	2,945.79	8.96	0.01	26
ε(BAO1), γ(TT + LSF2400)	2,946.20	9.37	0.01	26
ε(BAO1), γ(TT + LSF600)	2,946.52	9.69	0.00	26
ε(BAO1 + LSFEDGE), γ(TT)	2,948.33	11.50	0.00	26
ε(BAO1), γ(TT + NO1500)	2,950.52	13.68	0.00	26
ε(BAO1), γ(TT + NOEDGE)	2,950.69	13.86	0.00	26
ε(BAO1), γ(TT)	2,951.33	14.49	0.00	25
ε(BAO1 + NOEDGE), γ(TT)	2,951.73	14.90	0.00	26
ε(BAO1 + NO1500), γ(TT)	2,952.11	15.28	0.00	26
ε(BAO1), γ(TT + LSFMPS)	2,952.38	15.55	0.00	26
ε(BAO1), γ(TT + NO2400)	2,952.53	15.70	0.00	26
ε(BAO1 + NO600), γ(TT)	2,952.96	16.13	0.00	26
ε(BAO1 + LSF1500), γ(TT)	2,953.00	16.17	0.00	26
ε(BAO1), γ(TT + LSFEDGE)	2,953.20	16.37	0.00	26
ε(BAO1 + LSF2400), γ(TT)	2953.21	16.38	0.00	26
ε(BAO1 + NO2400), γ(TT)	2,953.22	16.39	0.00	26
ε(BAO1 + LSF600), γ(TT)	2,953.30	16.46	0.00	26
ε(BAO1 + LSFNP), γ(TT)	2,953.30	16.47	0.00	26
ε(BAO1 + LSFMPS γ(TT)	2,953.33	16.50	0.00	26
ε(BAO1), γ(LSFNP)	2,964.31	27.48	0.00	24

a BAO = Barred owl within the site in year *i*, BAO1 = barred owl within the site in year *i* + 1, NO600 = proportion of non-habitat in a 600-m circle, NO1500 = proportion of non-habitat in a 1,500-m circle, NO2400 = proportion of non-habitat in a 2,400-m circle, LSF600 = proportion of late-seral forest in a 600-m circle, LSF1500 = proportion of late-seral forest in a 1,500-m circle, LSF2400 = proportion of late-seral forest in a 2,400-m circle, LSFEDGE = amount of edge for late-seral forest in a 1,500-m circle, NOEDGE = amount of edge of non-habitat in a 1,500-m circle, LSFNP = number of patches of late-seral forest in a 1,500-m circle, LSFMPS = mean patch size of late-seral forest in a 1,500-m circle, TT = quadratic time effect.

b Post-hoc models formed by a combination of the top 2 models.

**Figure 2:**
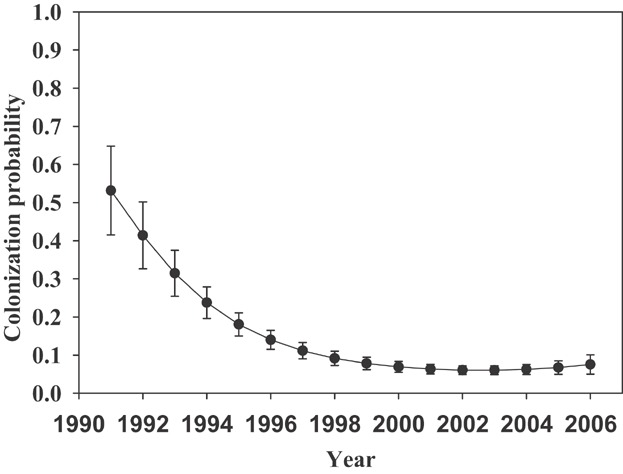
Year-specific colonization probability of spotted owl territories from model {ψ(.), ε(BAO1), γ(TT), *p*(T_1_ = T_2_ = …T_i_, BAO + t)}, Cle Elum Study Area, Washington, USA, 1989–2005, where ψ = probability of occupancy, ε = local extinction probability, γ = local colonization probability, p = detection probability, BAO = Barred owl within the site in year *i*, BAO1 = barred owl within the site in year *i* + 1, T = linear time effect, and TT = quadratic time effect. Error bars are +−1 SE.

When we added the barred owl covariates to our occupancy model for local extinction, we found that barred owl presence at a spotted owl territory was correlated with an increase in local extinction probability. The detection of a barred owl at a spotted owl territory in year *i* + 1 was correlated with an increase in local extinction in the interval between year *i* and year *i* + 1, as evidenced by the time-forward barred owl covariate (BAO1) showing the best fit among the barred owl covariates in the extinction models. Colonization probability showed a quadratic trend in which territory colonization declined rapidly during the early years of the study, and then declined more slowly in later years (Fig. 2). When we added habitat covariates to the extinction model, the only model that fit the data substantially better than a model with no habitat covariates was a model that provided weak evidence (ΔAIC = 2.99) for a decrease in local extinction with an increase in the amount of late-successional forest edge.

When we added habitat covariates to our colonization model, we found colonization probability decreased as the number of patches of late-successional forest increased (i.e., the late-successional forest became more fragmented).

Post-hoc models in which we included habitat covariates from the top 2 models for extinction and colonization indicated that local extinction probabilities declined as the amount of late-seral forest edge increased. The 95% confidence interval around the beta estimate for late-seral forest edge did not overlap 0 (Table2). Colonization probabilities declined as the amount of non-habitat increased, but the confidence interval for this parameter estimate barely overlapped 0 (γ = −2.46, 95% CI = −5.00–0.09; Table2).

**Table 2:** Logit-link parameter estimates, standard errors, and 95% confidence intervals for the model: {ψ(.), ε(BAO1 + LSFEDGE), γ(TT + NO600 + LSFNP), *p*(T_1_ = T_2_ =…T_*i*_, BAO + *t*)}^a^ for occupancy of spotted owl territories on the Cle Elum Study Area, Washington, USA, 1989–2005

			95% CI	
Parameter^b^	β	SE	Lower	Upper
ψ	0.63	0.44	−0.23	1.48
ε(Intercept)	−0.58	0.46	−1.47	0.31
ε(BAO1)	1.81	0.39	1.06	2.57
ε(LSFEDGE)	−0.18	0.08	−0.33	−0.02
γ(Intercept)	−1.11	0.39	−1.89	−0.34
γ(T)	−0.17	0.03	−0.23	−0.10
γ(TT)	0.02	0.01	0.01	0.04
γ(NO600)	−2.46	1.30	−5.00	0.09
γ(LSFNP)	−0.08	0.03	−0.14	−0.03

a ψ = probability of occupancy, ε = local extinction probability, γ = local colonization probability, *p* = detection probability.

b BAO = Barred owl within the site in year *i*, BAO1 = barred owl within the site in year *i* + 1, LSFEDGE = amount of edge for late-seral forest in a 1,500-m circle, T = linear time effect, TT = quadratic time effect, NO600 = proportion of non-habitat in a 600-m circle, and LSFNP = number of patches of late-seral forest in a 1,500-m circle.

## DISCUSSION

The barred owl range invasion in the Pacific Northwest has proceeded from north to south (Kelly et al. 2003), and the Cle Elum Study Area was likely populated with barred owls earlier than most study areas in Oregon. The proportion of spotted owl territories with barred owl detections on the Cle Elum Study Area has remained relatively constant or increased slightly during the study period, and we suspect that the effect of barred owls on the spotted owl population has followed a similar trend (constant or slightly increasing). Anthony et al. (2006) and Forsman et al. (2011) found some evidence that barred owl presence was negatively related to survival of spotted owls on the Cle Elum Study Area, which could explain why we found a positive relationship between the presence of barred owls and extinction probabilities. In our study, barred owl presence in year *i* + 1 (BAO1) was positively related to local extinction and barred owl presence in year i (BAO) was negatively related to detection. This indicates that barred owl presence may be negatively affecting both spotted owl territory occupancy and detectability. Olson et al. (2005) found either a positive effect on local extinction or a negative effect on colonization associated with barred owls on study areas in Oregon. Kroll et al. (2010) found barred owl presence had a positive effect on extinction probability for simple occupancy, and generally had a negative effect on spotted owl detection probabilities. Our findings add to the evidence that barred owls are having a negative influence on spotted owl populations (Courtney et al. 2004, Olson et al. 2005, Kroll et al. 2010, Forsman et al. 2011, Wiens et al. 2014).

### Habitat Composition

A negative relationship between the probability of colonization and the proportion of non-habitat within 600 m of the territory center is not surprising considering that most studies of habitat use by spotted owls indicate that they are usually associated with late-seral forest, and show a tendency to occupy territories with lower proportions of younger forest types (see Courtney et al. 2004 for review). Thus, one would expect territories with more non-habitat to be colonized at a lower rate than territories with more late-seral forest. However, we were surprised to find the amount of non-habitat was a better predictor of colonization than the amount of suitable habitat because the 2 variables are nearly the reciprocal of one another. We hypothesize that non-habitat is mapped more accurately than the other types, and thus, is more strongly correlated with colonization.

Irwin et al. (2004) was one of the few studies of spotted owl habitat associations that reported a lower proportion of old-forest (late-successional forest) in spotted owl core areas compared to random points on the landscape. Although Irwin et al. (2004) used some of the same territories for their study as we used in our study, our results seem contrary to theirs. This difference was possibly because Irwin et al. (2004) defined late-seral forest as stands with dominant trees >64 cm diameter at breast height, whereas we defined late-seral forest as closed-canopy stands with quadratic mean diameter of overstory trees >38 cm. Thus, our mapped late-seral forest included some stands that would not have been included in the Irwin et al. (2004) classification.

Our results indicate that forest fragmentation (indicated by number of late-seral forest patches) is negatively related to with spotted owl colonization probability. This was expected considering that most previous studies have found that measures of forest fragmentation were generally negatively related to vital rates of spotted owls (e.g., Franklin and Gutiérrez 2002, Courtney et al. 2004), positively related to spotted owl home range size (Carey et al. 1992), or more positively related to random points on the landscape than areas near spotted owl nests (Lehmkuhl and Raphael 1993, Morganti 1993, Hunter et al. 1995). The negative relation between forest fragmentation and owl fitness as measured above could be because higher fragmentation is related to lower abundance of some spotted owl prey (Wilson 2010, Lehmkuhl et al. 2006a, 2006b). Increased fragmentation may also negatively affect spotted owl social structure (Carey et al. 1992), thus leading to lower colonization probabilities.

We found a negative association between the amount of late-seral forest edge and the probability of local extinction. Spotted owl territory occupancy increases as the amount of late-seral forest edge increases, at least within the range of conditions that we studied. Other studies have also found positive relationships between spotted owl reproductive output and the amount of late-seral forest edge (Franklin et al. 2000, Olson et al. 2004). Franklin et al. (2000) speculated that the association between edge, reproduction, and survival of spotted owls in their study in northwestern California may have been related in part to the importance of the dusky-footed woodrat (*Neotoma fuscipes*) in the diet of spotted owls in that region. Zabel et al. (1995) also noted that spotted owls foraged near edges more than expected in their study area in northwestern California where woodrats were an important part of the diet. In northwest Oregon, where flying squirrels (*Glaucomys sabrinus*) are the main prey eaten by spotted owls, Glenn et al. (2004) found that spotted owls foraged disproportionately near edges between broadleaf and conifer forests, but not near edges between forests and non-forest (clear-cuts and early-seral vegetation on clear-cuts). Spotted owls in our study area may benefit from having at least some edge within their home ranges because, even though they feed mainly on flying squirrels that inhabit closed-canopy late-seral forests (Forsman et al. 2001, Lehmkuhl et al. 2006b), they also prey on species that thrive in openings or on edges between forests and openings, such as bushy-tailed woodrats (*Neotoma cinerea*), snowshoe hare (*Lepus americanus*), pika (*Ochotona princeps*), and gophers (*Thomomys talpoides*).

Franklin et al. (2000) and Olson et al. (2004) speculated that spotted owls may seek a balance between greater amounts of late-seral forest edge and lower fragmentation within their core areas. If spotted owls on our study area were assessing landscapes in a similar manner, it could explain the positive effect of late-seral forest edge and the negative effect of fragmentation in our study (see Fig. S1, available online at www.onlinelibrary.wiley.com, for examples of the relationship of different levels of non-habitat coverage and number of late-seral forest patches on estimates of colonization).

### Habitat Configuration

Although our results suggest that the amount of non-habitat within 600 m of the territory center exerts a stronger influence on colonization and extinction probabilities compared to larger scales, this does not mean that vegetative characteristics farther than 600 m from the territory center are not important. We examined only landscapes around nest or core locations and did not examine the much larger areas that spotted owls in Washington often use during winter when they tend to wander most extensively (Forsman et al. 2005, Hamer et al. 2007). These winter ranges contain much more late-seral forest than is contained within 600 m of the territory center, and probably provide critical resources for overwinter survival that are not found within the area immediately around the nest.

Our results support the recommendation that all analyses of spotted owls that include data collected by acoustic-lure surveys should include estimates of detection probabilities, preferably with an annual covariate that includes some measure of barred owl presence (Olson et al. 2005). In addition, the fact that our best model showed a relationship between the time-forward (BAO1) barred owl and spotted owl detection probabilities suggests that modeling barred owl presence at a spotted owl territory in a given year is too simplistic. Measures of barred owl presence should take into account the possibility of a lag effect on spotted owl detectability. Subsequent to our analysis, Bailey et al. (2009) recommended that occupancy analyses of northern spotted owls include per-visit detection probabilities for both spotted owls and barred owls. We did not model detection probabilities for barred owls in our analysis because multi-season models that included simultaneous estimation of detectability for multiple species were not available at the time. We may have underestimated the effects of barred owl presence on occupancy dynamics, but we believe that our approach was still valid because our barred owl covariate was based on multiple visits per year. If per-visit barred owl detection probabilities in our study area were similar to those estimated by Bailey et al. (2009), then the probability of detecting barred owls over 5 visits would be approximately 0.95, so any bias introduced by assuming barred owl detectability = 1.0 should be small.

The invasion of the barred owl has been identified as a potential threat to the northern spotted owl by nearly all researchers who have documented the range expansion of the barred owl in the Pacific Northwest (e.g., Taylor and Forsman 1976, Thomas et al. 1990, Dunbar et al. 1991, Hamer et al. 1994, Courtney et al. 2004). Based on the apparent negative relationship between barred owl presence and spotted owl occupancy in earlier studies such as Kelly et al. (2003), Dugger et al. (2005), and Olson et al. (2005), the USFWS is in the process of initiating studies in which barred owls will be removed (i.e., shot) from areas that were historically occupied by spotted owls (Buchanan et al. 2007, Livezey 2010, Diller 2013, USFWS 2013). If these experiments show a positive relationship between barred owl removal and spotted owl population growth, barred owl removal may be considered as a long-term management tool.

## MANAGEMENT IMPLICATIONS

On the east slope of the Cascade Range, where our study was conducted, loss of habitat from fire and insect outbreaks has been identified as a threat to the long-term viability of spotted owl populations, as well as a forest management challenge (Thomas et al. 1990, U.S. Department of Agriculture, Forest Service and U.S. Department of the Interior, Bureau of Land Management [USDA and USDI] 1994, Courtney et al. 2004, Gaines et al. 2010). Much of the concern stems from the high density of fire-intolerant tree species and continuous fuels present in many areas, a condition that is at least partially due to fire suppression (Camp 1999). Much of the Cle Elum Study Area is within late successional reserves, which were designated specifically for conservation of species like the spotted owl (USDA and USDI 1994). Management activities designed to reduce fuel continuity (e.g., forest thinning) in these late successional reserves may increase fragmentation by opening up the canopy of fire-prone stands, and create what we consider to be non-habitat for spotted owls. Although this may be an unavoidable situation for management agencies and land owners that are trying to meet multiple objectives, it is incumbent upon those agencies to do a thorough job of landscape planning so that areas treated to reduce the severity or spread of wildfire are carefully designed and placed so that they minimize the damage to habitats currently occupied by spotted owls. A recent exploration of treatment possibilities suggests that opportunities exist to achieve both fire and spotted owl management objectives (Gaines et al. 2010). In the long term, such treatments should also be designed with the objective of developing and maintaining closed canopy forests on those parts of the landscape that are thought to be least susceptible to wildfire, such as north aspects and watercourses. In light of the increased competition from barred owls described above, minimizing the effect of management activities on spotted owl habitat may be more critical now than before the barred owl invasion.
