# Supplementary material for: Barred owls and landscape attributes influence territory occupancy of northern spotted owls
Source: J Wildl Manage. 2014 Oct 9;78(8):1436–43. doi: 10.1002/jwmg.793 (PMC4277855; doi:10.1002/jwmg.793)
Supplement: Supplementary file 1 — Supporting Information. [file jwmg0078-1436-sd1.doc]

Supplemental Material

27 January, 2014

Influence Of Barred Owls And Landscape Attributes On Site Occupancy Of Northern Spotted Owls: in review.

Figure 1. Examples of estimated extinction (*ε* ) and colonization (*γ* ) parameters at different values of the landscape covariates from the best occupancy model {*ψ* (.), *ε* (BAO1 + LSFEDGE), *γ* (TT + NO600 + LSFNP), *p* (T_1_ =T_2_ =…T_i_, BAO + t)} for spotted owls on the Cle Elum Study Area, Washington, USA, 1989-2005. Each plot shows a 1500 m radius area around owl territory centers with a smaller 600 m radius circle within. Black pixels are late-seral forest, gray pixels are mid-seral habitat, white pixels are non-habitat. Panel A shows 2 territories with similar amounts of late-seral forest, but different amounts of LSFEDGE and corresponding differences in estimated *ε*. Panels B and C show pairs of territories with similar, relatively high (Panel B) and low (Panel C) estimates of *γ*, but with different values for NO600 and LSFNP. Acronyms in the model are as follows: BAO1 = Barred owl effect in year *y* + 1 correlating with colonization or extinction probability in the interval between *y* and *y* + 1; LSFEDGE = the distance (m) of late-seral edge /10,000 within 1500 m of a territory center; NO600 = the proportion of non-habitat within 600 m of a territory center; LSFNP = the number of patches of late-seral forest within the 1500 m r circle; t and T represent time and trend effects, respectively. The values for LSFEDGE, NO600, and LSFNP are averages across years for each territory, with a constant value for the other covariates in the model.


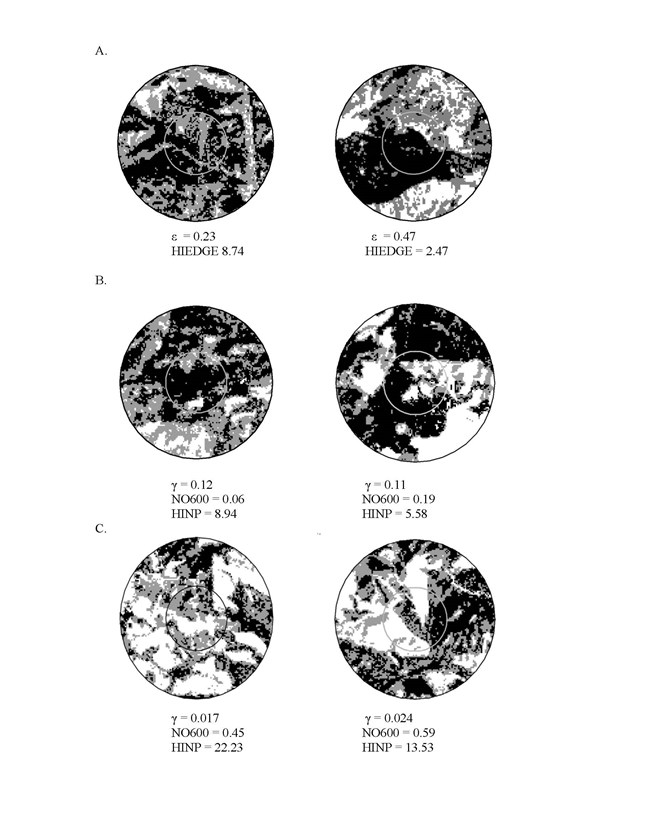


**Appendix A**. **Definitions of visit criteria and average maximum number of visits per year** **for site occupancy modeling of northern spotted owls on the Cle Elum Study Area**  **(adapted from Olson et al. 2005:932).**

**Visit Criteria**

1. Visits were considered “complete” if all calling points at a site (always > 1, the total number determined by the topography of each site) were covered during a night or day survey. Spot calls were conducted for ≥ 10 minutes at each point.

2. Trips taken to the same site ≤ 2 days apart were considered 1 visit. This usually occurred when an owl responded to a night survey call and a daytime follow-up trip was required to determine its reproductive status, or when the total number of calling points could not be surveyed in 1 trip.

3. If trips occurred 2 < days ≤ 7 apart then:

1. trips to the site in addition to those necessary to complete a visit were dropped
2. complete visits (according to protocol) within the 7-day interval were counted as separate visits.

4. Visits made to sites where pairs were established to count and/or band number of young could be counted as occupancy visits

**Average Maximum Number of Visits Per Year**

The average maximum number of visits for each year was determined after the visits for each site within a year were determined according to the visit criteria listed previously. The average maximum should be chosen so that at least 90% of the sites have that number of visits or fewer.

**Appendix B.** **Covariates used in modeling occupancy**

| PNEST | The proportion of owls that nested in a given year |
| --- | --- |
| FEC | Fecundity of female owls, assuming a 50:50 sex ratio among offspring |
| BAO | Barred owl within the site in year *i* |
| BAO1 | Barred owl within the site in year *i* + 1 |
| BAODIST | Barred owl detected within 0.8 km of the site center in year *i* |
| BAODIST1 | Barred owl detected within 0.8 km of the site center in year *i* + 1 |
| NO600 | Proportion of non-habitat in 600 m circle |
| NO1500 | Proportion of non-habitat in 1500 m circle |
| NO2400 | Proportion of non-habitat in 2400 m circle |
| LSF600 | Proportion of late-seral forest in 600 m circle |
| LSF1500 | Proportion of late-seral forest in 1500 m circle |
| LSF2400 | Proportion of late-seral forest in 2400 m circle |
| LSFEDGE | Amount of edge for late-seral forest in the 1500 m circle |
| NOEDGE | Amount of edge of non-habitat in the 1500 m circle |
| LSFNP | Number of patches of late-seral forest in the 1500 m circle |
| LSFMPS | Mean patch size of late-seral forest in the 1500 m circle |
